# Changing Patterns in Cancer Mortality from 1987 to 2020 in China

**DOI:** 10.3390/cancers15020476

**Published:** 2023-01-12

**Authors:** Binbin Su, Panliang Zhong, Yundong Xuan, Junqing Xie, Yu Wu, Chen Chen, Yihao Zhao, Xinran Shen, Xiaoying Zheng

**Affiliations:** 1 School of Population Medicine and Public Health, Chinese Academy of Medical Sciences & Peking Union Medical College, Beijing 100730, China; 2 Department of Urology, The Third Medical Centre, Chinese PLA (People’s Liberation Army) General Hospital, Beijing 100853, China; 3 Centre for Statistics in Medicine, NDORMS, University of Oxford, Oxford OX3 7LD, UK

**Keywords:** cancer mortality, long-term trends, China, urban-rural disparity, age-period-cohort effects

## Abstract

**Simple Summary:**

We analyzed the long-term mortality trends and their Age-Period-cohort effects for overall cancer and its 10 sub-types in China. The results show that the overall cancer ASMR has declined, but the absolute cancer cases kept increasing due to the growing elderly population, with the death rates for almost all types of cancer among older Chinese continued to rise during the past three decades. The birth cohort RRs peaked in 1920–1930 and tended to decline in successive cohorts for most cancers except for leukemia, lung cancer in rural, and breast and cervical cancer in females, whose relative risks were rising in the very recent cohorts. The rising mortality related to lung, colorectal, breast and cervical cancer and the increasing burden of cancer death in rural areas should receive higher priority in managing cancer burden and calls for targeted public health actions to reverse the trend.

**Abstract:**

Background: China has the highest number of new cancer cases and deaths worldwide, posing huge health and economic burdens to society and affected families. This study comprehensively analyzed secular trends of national cancer mortality statistics to inform future prevention and intervention programs in China. Methods: The annual estimate of overall cancer mortality and its major subtypes were derived from the National Health Commission (NHC). Joinpoint analysis was used to detect changes in trends, and we used age-period-cohort modeling to estimate cohort and period effects in Cancers between 1987 and 2020. Net drift (overall annual percentage change), local drift (annual percentage change in each age group), longitudinal age curves (expected longitudinal age-specific rate), and period (cohort) relative risks were calculated. Results: The age-standardized cancer mortality in urban China has shown a steady downward trend but has not decreased significantly in rural areas. Almost all cancer deaths in urban areas have shown a downward trend, except for colorectal cancer in men. Decreasing mortality from cancers in rural of the stomach, esophagus, liver, leukemia, and nasopharynx was observed, while lung, colorectal cancer female breast, and cervical cancer mortality increased. Birth cohort risks peaked in the cohorts born around 1920–1930 and tended to decline in successive cohorts for most cancers except for leukemia, lung cancer in rural, and breast and cervical cancer in females, whose relative risks were rising in the very recent cohorts. In addition, mortality rates for almost all types of cancer in older Chinese show an upward trend. Conclusions: Although the age-standardized overall cancer mortality rate has declined, and the urban-rural gap narrowed, the absolute cancer cases kept increasing due to the growing elderly population in China. The rising mortality related to lung, colorectal, female breast, and cervical cancer should receive higher priority in managing cancer burden and calls for targeted public health actions to reverse the trend.

## 1. Introduction

Cancer is a severe public health issue in China [1]. GLOBOCAN 2020 estimated that there were 9,958,133 cancer deaths globally in 2020, and the number of cancer deaths in China reached 3 million, accounting for 30.2% of all cancer deaths worldwide and ranking first worldwide [2]. China’s official figures show that the age-standardized mortality rate (ASMR) of all cancers was 105.19 per 100,000 [3], cancer has already become the first leading cause of death in China.

Cancer caused an estimated 62.9 million disability-adjusted life years (DALYs) in China in 2017 [4]. Lung cancer accounts for the largest proportion, followed by liver cancer, gastric cancer, esophageal cancer, and other digestive system tumors [5]. It has been widely reported that cancer mortality continues to increase in China due to the aging population and increasing adoption of carcinogenic behaviors [2,6]. The level and patterns of cancer motility differed significantly according to cancer types, genders and areas [7,8]. Previous studies have reported trends in cancer mortality in China for specific populations [9], different regions and time periods [10]. These studies found selected cancers reported increasing trends for cancers of the lung [11], breast [12], colorectal [13], and stomach [14]. However, there is no consistent conclusion regarding the trend of cervical cancer deaths in China [15,16].

Although China’s national level mortality data for the period 1987–1999 and 1987–2009 have been reported in previous studies [17,18], China has experienced considerable changes in the last decade. For example, China’s new health-care reform policy was initiated in 2009. Since then, China has launched a package of programs to improve the health of the population and disease control, such as expanding health insurance coverage, increasing investment in health care, and providing health education [19], which has had a significant impact on reducing cancer deaths. And China’s GDP overtook Japan to become the world’s second-largest economy in 2010 and hit 101.5986 trillion yuan ($15.51 trillion) in 2020 [20], exceeding the 100 trillion yuan mark for the first time, etc. As a result, China’s cancer spectrum is shifting from a developing country to a developed country spectrum [21]. Therefore, it is of great significance to conduct a systematic and up-to-date analysis of cancer mortality in China. The main objective of this study is to provide empirical basis for the optimization of public health policies and cancer prevention and control strategies of the Chinese government.

## 2. Materials and Methods

Information on overall cancer and its major sub-types mortality by age from 1987 to 2020 was derived from China’s National Health Commission (CNHC) death registration system. which was considered a main authoritative data source to provide the most representative information on national mortality trends and patterns. The death data from five administrative organizations [22] (including the death medical certificate information, the total population information, the registered permanent residence cancellation information, the cremation information, and the termination of social security information) were incorporated after the IDs quality control and removal of duplication, all categories were coded according to the International Classification of Diseases (ICD), the 9th Revision (ICD-9) and the 10th Revision (ICD-10). ICD-9 was operated on before 2002, and ICD-10 thereafter. As with most mortality reporting data, this dataset suffers from the same underreporting issues. According to previous research, the underreporting rate for this data was roughly 2% in 2017 [23].

The ASMR by areas and sexes were calculated based on the World Standard Population (WSP) [24] using direct calculation method. To calculate the annual variation in mortality rates and identify significant change points, we used joinpoint regression models to identify trends in cancer mortality over time, using the Joinpoint software Version 4.9.1.0 (Statistical Research and Applications Branch National Cancer Institute, Washington, MD, USA) [25]. In this study, joinpoint analysis was used to identify years (as the independent variable) with significant changes in mortality rate over the study period and the size of these changes (as the percentage change in rate per year). Using a natural log-linear model enables the analysis of a constant percentage change in mortality rates over time.

A age-period-cohort (APC) model was used to establish independent estimates of the age, period and cohort effects on cancer mortality [26]. In the APC model, Net-drift and Local-drift were calculated separately, of which, Net-drift denotes the time-trend effect of the whole population, while Local-drift indicates the log-linear trend for each age group [26,27]. The longitudinal age curve, period relative risks and cohort relative risks were displayed simultaneously in this paper. Successive 5-year age intervals (from 0 to 5 years to 85 years and older) were used to calculate the longitudinal age curve. The period effects are the age-specific relative risk ratio, with 2001 to 2005 as a reference. The cohort effects are the age-specific risk ratio of the 14 cohorts among individuals, including those born from 1906–1910 (median, 1908) to 2016–2020 (median, 2018), with the birth cohort of 1961–1965 (median, 1963) as a reference. Those parameters were estimated by using the Web-based APC tool developed by the U.S. National Cancer Institute [28].

## 3. Results

### 3.1. Long-Term Trends in Cancers Mortality between1987 and 2020

**Figure 1:**
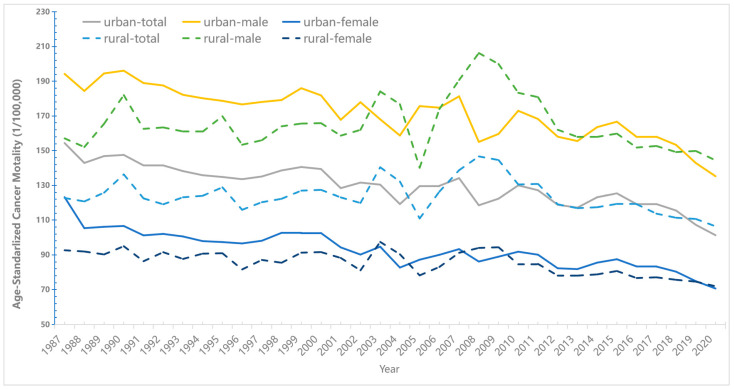
Trends in Age-Standardized Cancer Mortality in urban and rural China by sex: 1987–2020.

Figure 1 shows the trend age-standardized cancer mortality in China from 1987–2020. The age-standardized mortality manifested a downward trend. Lung, liver, stomach, colorectal, and esophageal cancer were China’s five most fatal cancers (Supplementary Figure S1). For a long time, the overall cancer ASMR was higher in urban than in rural, but in recent years this trend was narrowed. And we can see a more pronounced upward trend in cancer mortality in rural areas of China between 2007 and 2011, as can also be seen in Supplementary Table S1.

In 2020, the cancer ASMR was 135.26 per 100,000 for urban males and 70.66 per 100,000 for urban females. By 2020, the ASMR of cancer in urban areas reached 101.32 per 100,000, with 135.26 per 100,000 for urban men and 70.66 per 100,000 for urban women. Those figures amounted to 194.25 and 123.21 per 100,000 people respectively in 1987. Meanwhile, the cancer mortality in rural China was 106.45 per 100,000, with 144.44 per 100,000 for urban males and 71.96 per 100,000 for urban females in 2020. The ASMR of overall cancer declined by 34.4% and 17.5%, respectively in urban and rural areas during the study period in China.

The results of the Joinpoint analysis are presented in Table 1. For the rural population, men generally have higher cancer ASMR than women. Lung cancer is the largest cause of cancer death in both areas and sexes. with a rate of 30.26 per 100,000 in rural in 2020. Cancers such as nasopharyngeal, liver, oesophagus, stomach, cervical and leukemia have shown a significant decline over the last 30 years. Looking at the different sub-periods of joinpoint regression analysis, the overall cancer mortality rate kept stable in rural males. While urban areas presented a significant downward trend, with a significant decline of −0.8% per year. In males, declining mortality trends for nasopharynx, stomach, colorectal and liver cancers have been observed over the three decades, except bladder cancer and leukemia. However, mortality from lung cancer in rural males has shown a significant upward trend between 1987 and 2005, although no significant increase has been observed in the recent decade. For females, downward trends in mortality have been observed for all cancers over the last three decades, except for breast and lung cancer. From 1993–2002, the mortality rate from breast cancer was rising at a rate of 5.3% per year, at the same time, the mortality rate for cervical cancer showed a relatively rapid upward trend over the period 2005–2020, with an increase by +7.2% per year.

For the urban population, the overall ASMR were continuous decreasing for both sexes. And the mortality was decreased in both sexes for most cancer sites, with the largest drop in esophagus cancer of −3.6% per year. Nasopharynx, esophagus, and stomach cancer sites have also decreased significantly in recent years. Although a minor and insignificant decline trend of cervix uteri cancer mortality was observed in urban females, we found marked and contrasting trends in cervical cancer mortality among urban women in the following two periods, 1994–2008 (−2.4% per year) and 2008–2016 (+99% per year), respectively. And a similar trend has been observed in rural populations.

### 3.2. The Overall and Age-Specific Annual Percentage Change of Cancer Mortality

**Figure 2:**
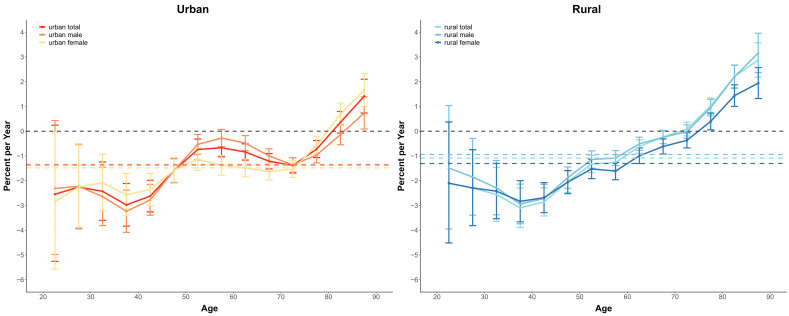
Local drift with net drift values for Cancer mortality and sex difference by area in China from 1987 to 2020. (Net drift represents the overall annual percentage change. Local drift values represent the annual percentage change in each age group).

The Net-drift and Local-drift for overall cancer and specific cancer diseases are presented in Figure 2. Net-drift refers to the overall annual average trend in the whole population over the study period, whereas the Local-drift indicates the average annual trend for different age groups over the study period. (Figure 2 and Supplementary Figure S2). In this study, the rural and urban Net-drift shows similar trends and characteristics with values significantly less than zero, indicating an overall downward trend in cancer mortality in China across the study period.

There was little reduction in the overall cancer mortality in the urban population, whereas for the rural, the trend was smaller but still favorable, with a decline rate of −1.26% (95% CI, −1.63 to −0.88) and −1.55% (95% CI, −1.96 to −1.13) respectively. The gender differences in cancer mortality was also significant, with less improvement in men than in women (−1.53% (95% CI, −1.94 to −1.12) versus −1.64% (95% CI, −2.06 to −1.22). and also less improvement in urban (−1.09% (95% CI, −1.47 to −0.71)) than in rural (−1.38% (95% CI, −1.77 to −1.00)). The local-drift of values lies predominantly below 0 for most age groups, indicating significant improvements in Chinese Cancer mortality control. Except the 45–75 years old of male population in rural (0.01% to 0.25%), 75+ years of rural female (0.39% to 1.43%), 45–85 years of urban female (0.03% to 0.20%) and 85+ years of urban male 0.73%).

In terms of specific cancer types, the local drift analysis results are consistent with the above pattern, indicating that China has made some achievements in cancer mortality prevention and control. The greatest improvements were esophageal cancer for rural women 25 to 60 years of age (−6.2%/year to 11.5%/year) and bladder cancer of 25 to 44 years of age for rural women (−7.42%/year to −6.76%/year). However, there are increasing trends in breast cancer among women in rural areas and cervical cancer among women in urban areas, lung cancer in the over-45 group in rural areas, leukemia in the over-50 group in rural areas. At the same time, it is necessary to note that the mortality rate for almost all types of cancer in China’s elderly population (over 70 years of age) has shown an increasing trend over the past three decades.

### 3.3. Age-Period-Cohort Effects on Cancer Mortality

**Figure 3:**
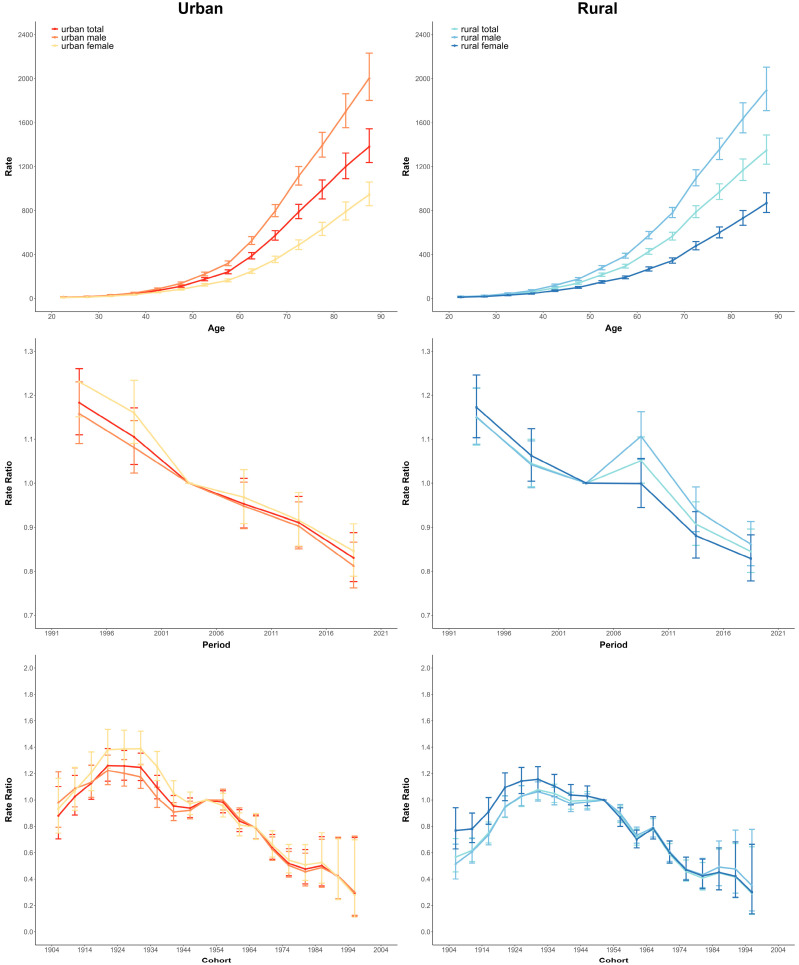
Parameter estimates of age, period, and cohort effects on Cancer mortality rate in China from 1987 to 2020.

Figure 3 shows estimates of age, period, and cohort effects on cancer. The age effects showed an expected exponential distribution. Cancer mortality rates in rural and urban populations increase rapidly with age, and the rate of increase is higher in males than in females, and higher in urban than rural areas. Period effects tended to show differing directions across urban and rural areas. For urban, the period effects showing a steady downward trend over time in both sexes, suggesting significant enhancements throughout the study period. But for rural, although the risk of cancer death in rural areas showed a downward trend, there were some fluctuations between 2005 and 2010.

Cohort effects showed similar patterns between urban and rural areas in both sexes. The most notable improvements across the birth cohorts were observed among Chinese women living in urban areas, with a progressive improvement in mortality among persons born from 1930 onwards. But there has been little progress for the older cohorts born between 1905 and 1935, with the birth cohorts (1930–1935) of both sexes in urban and rural areas tending to achieve their maximum risk.

Supplementary Figure S3 summarize the age-period-cohort effects and their changing patterns by different cancer sites according to sex and area. In rural areas, the temporal developmental stages of most cancers are obviously similarity in terms of sex, and the birth cohorts effects of colorectal, anal, nasopharynal, and stomach cancer reached their maximum risk in the cohort of 1925–1930. But the breast and lung cancer’s death risk has increased in recently born generations.

This pattern continues in urban areas. For the urban population, the maximum cohort risks of colorectal and anus, liver, nasopharynx, esophagus, and stomach cancer peaked in the generations born from 1925 to 1930 regardless of sex and then continued to decline (Supplementary Figure S3). However, there is an increasing risk of lung cancer among birth cohorts before 1970 for rural populations. In particular, a rising risk of cervix, breast, leukemia and colorectal cancer has also been observed in the recent decade.

## 4. Discussion

This study provides an updated and comprehensive analysis of time trends in cancer mortality in China over the past three decades, focusing on different trends and age-period-cohort effects between rural and urban regions. According to the GLOBOCAN database, the cancer mortality rate in China is 163.9/100,000 for males and 98.1/100,000 for females, making China’s cancer mortality rate relatively low and one of the 57 countries with the lowest mortality rates in the world [2]. This data is generally consistent with this study’s data extracted from the CNHC. And the ASMR in both rural and urban china has shown a downward trend, indicating a definitive effect of cancer mortality control in China in recent decades.

In both 1987 and 2020, the top three cancers in terms of mortality in urban areas were lung cancer, liver cancer and stomach cancer. However, the spectrum of cancer deaths in rural areas has changed, with the top three cancers in 1987 being stomach, liver and oesophagus cancer, but in 2020 the top three cancers became lung, liver and stomach cancer.

There were striking differences between rural and urban areas in both mortality rates and time trends. In 2020, The Age-standardized mortality rates for the nasopharyngeal, oesophageal, liver, stomach, and cervical cancers were all higher in rural areas than in urban areas. However, the mortality rates for colorectal and breast cancers are higher in urban areas. Regarding temporal trends, death rates from all cancers in urban areas show an upward trend, except for nasopharyngeal and gastric cancers, which show a downward trend. However, only colorectal cancer in men showed a slight upward trend after age-standardization. In rural areas, the ASMR of lung cancer in rural areas, and colorectal cancer in males showed an upward trend. Differences in socio-economic development, health service levels and lifestyle habits between urban and rural areas have probably influenced cancer mortality patterns in rural and urban China [29,30].

Lung cancer has emerged as the largest cancer burden in China, both in urban and rural areas. In recent years, lung cancer mortality has continued to rise, especially in rural areas. While it has decreased gradually in the United states and some other western countries [10]. Increasing tobacco consumption [31] and outdoor air pollution [32,33] may be important causes. In terms of cohort effects, the cohort patterns of lung cancer show that RRs peaked in generations born from 1925 to 1930 and declined among successive cohorts.

There were similar trends in liver cancer mortality between urban and rural populations. And liver cancer has long been the top three causes of cancer deaths in rural and urban China. It is estimated that 0.82 million liver cancer deaths occurred worldwide in 2020, and China is believed to account for nearly half (0.39 million) [2]. However, the age-specific standardized mortality rates for liver cancer showed a consistent and steady downward trend in recent years. The cohort effects of liver cancer peaked in generations born from 1925 to 1930 and decreased among the successive cohorts of both sexes in rural and urban China. HBV and aflatoxins have been proved as major risk factors, which work individually and synergistically in liver cancer etiology [34,35].

The mortality rate of stomach cancer also shows significant urban-rural differences, with rural areas being higher than urban areas and men higher than women. According to statistics, Chinese stomach cancer deaths reached 370,000 in 2020 [2]. However, stomach cancer mortality has decreased substantially in China, this is similar to the global trend of stomach cancer deaths in most countries [36]. Urban areas have declined more rapidly in China. And China’s stomach cancer mortality was lower than that in Japan [37]. The results of the APC model show that a promising trend in mortality from stomach cancer has been observed in both sexes in both urban and rural areas, particularly among successive birth cohorts born in 1920. It is estimated that 60% of stomach cancers worldwide were associated with chronic infection with Helicobacter pylori [38]. Other risk factors include tobacco use, dietary habits and food preservation methods, and availability of fresh fruits and vegetables. Much of the improvement in stomach cancer mortality in China may be due to the improvements in lifestyle habits and risk factors.

Colorectal cancer is one of the most frequently diagnosed malignancies globally [39], Our study found that the mortality rate of colorectal cancer in China was higher in urban areas than in rural areas and higher in men than in women. There were also gender differences in trend, with mildly increasing mortality rates observed in males. This increase may be due to western eating habits [40], unhealthy health behaviors [41], and the deepening ageing process [42].

Compared with the urban area, the mortality of esophageal cancer is much higher in rural areas. And some previous studies have reported similar conclusions [43]. Mortality from esophageal cancer has declined steadily for both sexes in rural and urban China, and the decline was most marked in rural areas, which is also reflected in cohort effects and successive declines in risk in all generations born after 1920. In China, 90% of nasopharyngeal cancers are esophageal squamous cell carcinoma (ESCC) [44]. Low intake of fresh fruit and vegetables, Hot beverage and pickled vegetables, HPV Infections, and Genetic changes were considered to be the main risk factors for ESCC [45].

In recent years, the global mortality rate from breast cancer has risen rapidly and has replaced lung cancer as the world’s largest cancer burden in 2020 [2]. The disease burden of breast cancer in China is also increasing, with an estimated 1.6 million diagnoses and 1.2 million deaths due to breast cancer each year [46]. In this study, the age-standardized mortality rates of breast cancer increased significantly among rural females, but have slowly declined in urban areas, which is generally consistent with some previous findings [47]. High prevalence of risk factors but poor diagnostic techniques may be an important reason. For example, the first-stage detection rate of breast cancer in China is 13.5, but it reaches 50.5% in developed countries such as the United States [48]. This has led to the low survival period of breast cancer in China, especially in rural areas where medical resources are scarce. Changes in living and eating habits may also be an important reason affecting the death trend of breast cancer in China [46].

There has been a slight decline in leukemia mortality over the last three decades. And the RRs reached the highest level in the birth cohort between 1930 and 1950 and showed a downward trend in the following birth cohorts of both genders in rural and urban China. Leukemia was the leading cause of childhood and adolescent cancer in China, as well as in the United States and the United Kingdom [8]. In addition to the group of children and adolescents, the leukemia-related mortality rate among seniors in China is also increasing. The causes of leukemia have not been systematically established, but ionizing radiation and levels of maternal education have been shown to be associated with leukemia, and further research is needed.

A declining trend in bladder cancer mortality has been observed in Chinese populations during the whole period in both areas, the risk ratio of death from bladder cancer peaked in the 1920–1925 birth cohort and decreased among the successive cohorts. In most countries, tobacco use and exposure to hazardous chemicals are the major risk factors for bladder cancer [49]. In China, however, the main risk factors are the changing dietary habits [50] and chemical substances like benzidine [51]. With the rapid industrialization in China, the exposure to occupational carcinogens may increase. Moreover, the high smoking rates make bladder cancer continues to be a severe health problem in china.

Nasopharyngeal carcinoma (NPC) is a cryptic malignant tumor with marked racial and geographic differences. NPC is particularly prevalent in southeastern provinces of China [52]. Mortality from NPC is higher in China’s urban areas than in rural areas, and had been favorable for both sexes during the whole study period. A decreasing risk was observed among birth cohorts born after the 1910s. NPC was once considered endemic in the southern part of China. And the global data from GLOBOCAN in 2020 showed that 26% of all new deaths of NPC were registered in China [53]. EBV infection and smoking as well as specific dietary habits (e.g., salted fish) were considered to be the major risk factors for nasopharyngeal carcinoma [54,55,56].

For cervix cancer, the mortality in China shows fluctuating changes over the study period in both areas. A clear downward trend was observed in the period 1987–2008 among the urban female population, followed by a significant upward trend in 2008–2016. A similar trend was also identified in rural women. This is consistent with some of the previous research findings in China [57].

To conclude, this study provides a comprehensive overview on the mortality patterns of selected cancers in China over the past three decades. China has made a remarkable improvement in the control of cancer deaths among rural and urban populations, with the vast majority of cancer deaths show a downward trend, but the cancer burden has increased in the elderly population and in some specific cancer cites, and a relatively high rate of cancer deaths in rural areas of China was observed. In addition, the age-standardized 5-year relative survival was merely 40.5% during 2012–2015 [58]. There was still a large gap compared to developed countries [59]. Comprehensive measures including tobacco control, improving the access to health services and air condition and living environment were urgently needed.

## 5. Conclusions

Although the age-standardized overall cancer mortality rate has declined, and the urban-rural gap narrowed, the absolute cancer cases kept increasing due to the growing elderly population in China. The rising mortality related to lung, colorectal, female breast and cervical cancer should receive higher priority for the government in managing cancer burden and calls for targeted public health actions to reverse the trend.

## Figures and Tables

**Table 1:** Joinpoint analysis of Age-Standardized Mortality Rates from Cancers in urban and rural areas.

Cancer Site		Mortality Rate (per 100,000) ^a^		Total Study Period ^b^		Period 1		Period 2		Period 3		Period 4		Period 5	
		1987	2020	AAPC (%)	95% CI	Years	APC (%)	Years	APC (%)	Years	APC (%)	Years	APC (%)	Years	APC (%)
Cancers in urban areas	total	154.38	101.32	−0.8 *	(−1.0,−0.7)	1987–2018	−0.7 *	2018–2020	−7.3	—	—	—	—	—	—
	male	194.25	135.26	−0.8 *	(−0.9,−0.6)	1987–2017	−0.6 *	2017–2020	−4.8	—	—	—	—	—	—
	female	123.21	70.66	−1.0 *	(−1.2,−0.9)	1987–2018	−0.9 *	2018–2020	−6.8	—	—	—	—	—	—
Nasopharyngeal	total	2.44	0.84	−2.9 *	(−3.3,−2.5)	1987–2020	−2.9 *	—	—	—	—	—	—	—	—
	male	3.12	1.3	−2.6 *	(−3.0,−2.2)	1987–2020	−2.6 *	—	—	—	—	—	—	—	—
	female	1.63	0.39	−3.6 *	(−4.1,−3.1)	1987–2020	−3.6 *	—	—	—	—	—	—	—	—
Esophageal	total	13.97	6.2	−1.7 *	(−2.2,−1.3)	1987–2000	−4.0 *	2000–2005	6.8	2005–2008	−16.3	2008–2015	4.1	2015–2020	−6.3 *
	male	18.96	9.85	−1.5 *	(−1.9,−1.1)	1987–2001	−3.0 *	2001–2005	7.7	2005–2008	−14.1	2008–2015	3	2015–2020	−5.7 *
	female	8.46	2.84	−2.5 *	(−3.2,−1.9)	1987–1997	−6.8 *	1997–2005	3.1	2005–2008	−20.5	2008–2015	6.5 *	2015–2020	−7.5
Stomach	total	28.82	10.54	−2.4 *	(−2.7,−2.1)	1987–2020	−2.4 *	—	—	—	—	—	—	—	—
	male	37.76	15.29	−2.4 *	(−2.7,−2.1)	1987–2020	−2.4 *	—	—	—	—	—	—	—	—
	female	18.75	6.27	−2.6 *	(−2.9,−2.4)	1987–2020	−2.6 *	—	—	—	—	—	—	—	—
Colon, rectum & anus	total	10.65	9.22	−0.1	(−0.4,0.1)	1987–2020	−0.1	—	—	—	—	—	—	—	—
	male	10.36	11.79	0.4 *	(0.1,0.6)	1987–2000	1.1 *	2000–2004	−5.0 *	2004–2007	11.5 *	2007–2012	−3.3 *	2012–2020	0.7
	female	10.49	6.96	−0.8 *	(−1.0,−0.5)	1987–1989	−8.3	1989–2000	0.6	2000–2003	−9.2	2003–2007	6.0	2007–2020	−1.6 *
Liver	total	24.45	13.78	−1.2 *	(−1.6,−0.9)	1987–2020	−1.2 *	—	—	—	—	—	—	—	—
	male	33.82	20.57	−1.1 *	(−1.5,−0.8)	1987–2005	−0.3	2005–2020	−2.3 *	—	—	—	—	—	—
	female	13.79	7.26	−1.4 *	(−1.8,−1.1)	1987–2020	−1.4 *	—	—	—	—	—	—	—	—
Lung	total	40.36	29.79	−0.4 *	(−0.6,−0.1)	1987–2000	0.6	2000–2003	−6	2003–2015	1.1 *	2015–2020	−3.9 *	—	—
	male	52.47	43.92	−0.2 *	(−0.4,−0.0)	1987–2000	0.9 *	2000–2003	−7.1	2003–2006	4.1	2006–2017	0.1	2017–2020	−5.4 *
	female	26.95	16.96	−0.7 *	(−1.0,−0.4)	1987–2000	0.5	2000–2004	−7.8	2004–2010	4.3	2010–2020	−2.3 *	—	—
Breast	total	3.85	2.96	−0.4 *	(−0.6,−0.1)	1987–1996	−0.5	1996–1999	4.7	1999–2002	−6	2002–2017	0.3	2017–2020	−4.9
	male														
	female	7.56	5.69	−0.4 *	(−0.6,−0.1)	1987–2020	−0.4 *	—	—	—	—	—	—	—	—
Cervix	total	2.86	1.41	−0.6	(−1.7,0.5)	1987–1994	−10.1 *	1994–2008	−2.4 *	2008–2016	9.9 *	2016–2020	−6.8	—	—
	male														
	female	5.48	2.78	−0.4	(−1.5,0.7)	1987–1994	−9.9 *	1994–2008	−2.5 *	2008–2016	11.0 *	2016–2020	−7.4	—	—
Bladder	total	2.66	1.45	−1.6 *	(−1.9,−1.2)	1987–1999	0.1	1999–2002	−9.3	2002–2020	−0.6	—	—	—	—
	male	3.97	2.48	−1.6 *	(−1.9,−1.2)	1987–2000	0.3	2000–2003	−15.2 *	2003–2006	8.2	2006–2020	−1.4 *	—	—
	female	1.31	0.61	−2.0 *	(−2.4,−1.6)	1987–2020	−2.0 *	—	—	—	—	—	—	—	—
Leukemia	total	4.46	2.68	−0.9 *	(−1.1,−0.6)	1987–1996	−2.6 *	1996–1999	4.9	1999–2004	−3.3	2004–2007	4	2007–2020	−2.0 *
	male	5.04	3.15	−0.7 *	(−0.9,−0.4)	1987–1996	−2.7 *	1996–1999	6.7	1999–2004	−4.3	2004–2007	5.7	2007–2020	−2.0 *
	female	3.82	2.24	−1.1 *	(−1.3,−0.8)	1987–2020	−1.1 *	—	—	—	—	—	—	—	—
Cancers in rural areas	total	122.74	106.45	−0.2	(−0.5,0.1)	1987–2009	0.4 *	2009–2020	−1.9 *	—	—	—	—	—	—
	male	157.09	144.44	−0.1	(−0.4,0.2)	1987–2005	0.1	2005–2008	6.1	2008–2020	−2.8 *	—	—	—	—
	female	92.67	71.96	−0.6 *	(−0.8,−0.4)	1987–2009	−0.2	2009–2020	−1.7 *	—	—	—	—	—	—
Nasopharyngeal	total	2.22	1.14	−1.9 *	(−2.4,−1.4)	1987–2020	−1.9 *	—	—	—	—	—	—	—	—
	male	3.28	1.72	−1.5 *	(−2.1,−1.0)	1987–2020	−1.5 *	—	—	—	—	—	—	—	—
	female	1.26	0.59	−2.6 *	(−3.2,−2.0)	1987–2020	−2.6 *	—	—	—	—	—	—	—	—
Esophageal	total	22.93	8.16	−2.9 *	(−3.7,−2.2)	1987–2008	−1.1	2008–2020	−7.0 *	—	—	—	—	—	—
	male	29.99	12.95	−2.3 *	(−3.0,−1.6)	1987–2008	−0.6	2008–2020	−6.1 *	—	—	—	—	—	—
	female	16.58	3.80	−4.3 *	(−5.1,−3.6)	1987–2008	−2.3 *	2008–2020	−8.7 *	—	—	—	—	—	—
Gastric cancer	total	27.37	12.45	−2.2 *	(−2.6,−1.8)	1987–2008	−1.1 *	2008–2020	−4.7 *	—	—	—	—	—	—
	male	36	18.21	−1.9 *	(−2.3,−1.5)	1987–2008	−0.9 *	2008–2020	−4.3 *	—	—	—	—	—	—
	female	19.58	7.26	−2.8 *	(−3.1,−2.4)	1987–2006	−1.6 *	2006–2020	−4.7 *	—	—	—	—	—	—
Colon, rectum & anus	total	7.21	7.6	0.4 *	(0.0,0.7)	1987–2020	0.4 *	—	—	—	—	—	—	—	—
	male	7.93	9.63	0.6 *	(0.3,0.9)	1987–1992	−2	1992–2006	1.0 *	2006–2009	6.3	2009–2013	−6.6	2013–2020	3.6 *
	female	6.67	5.79	−0.3 *	(−0.6,−0.0)	1987–2004	−0.9 *	2004–2009	3.60	2009–2012	−7.9	2012–2020	2.3 *	—	—
Liver cancer	total	24.66	17.64	−1.2 *	(−1.6,−0.7)	1987–2003	0.9 *	2003–2020	−3.0 *	—	—	—	—	—	—
	male	35.98	26.75	−1.0 *	(−1.4,−0.6)	1987–2002	1.0 *	2002–2005	−5.8	2005–2008	4.9	2008–2020	−3.9 *	—	—
	female	14.14	8.92	−1.6 *	(−2.0,−1.2)	1987–2000	1.0 *	2000–2020	−3.0 *	—	—	—	—	—	—
Lung cancer	total	15.55	30.26	2.4 *	(2.1,2.8)	1987–2009	3.2 *	2009–2020	0.4	—	—	—	—	—	—
	male	22.96	44.37	2.3 *	(1.9,2.6)	1987–2005	2.3 *	2005–2008	9.7	2008–2020	−0.8	—	—	—	—
	female	8.94	17.46	2.4 *	(2.1,2.8)	1987–2020	2.4 *	—	—	—	—	—	—	—	—
Breast cancer	total	1.98	2.43	1.2 *	(0.7,1.6)	1987–1996	0.6	1996–2002	6.1	2002–2005	−13.3	2005–2008	13.5	2008–2020	−0.5
	male														
	female	3.94	4.69	1.0 *	(0.6,1.5)	1987–1993	−1.1	1993–2002	5.3 *	2002–2005	−12.2	2005–2008	11.2	2008–2020	−0.8
Cervical carcinoma	total	3.97	1.81	−2.0 *	(−3.2,−0.8)	1987–2001	−3.1 *	2001–2006	−14.4	2006–2020	6.9 *	—	—	—	—
	male														
	female	7.82	3.53	−1.9 *	(−3.1,−0.6)	1987–1990	−17.1	1990–2001	−0.1	2001–2005	−21.7	2005–2020	7.2 *	—	—
Bladder cancer	total	1.11	1.33	0.2	(−0.2,0.5)	1987–2020	0.2	—	—	—	—	—	—	—	—
	male	1.85	2.34	0.1	(−0.3,0.5)	1987–2020	0.1	—	—	—	—	—	—	—	—
	female	0.5	0.5	−0.9 *	(−1.4,−0.3)	1987–2020	−0.9 *	—	—	—	—	—	—	—	—
Leukemia	total	3.30	3.03	−0.4 *	(−0.5,−0.2)	1987–2020	−0.4 *	1987–2005	−0.8 *	2005–2008	4.40	2008–2020	−1.5 *	—	—
	male	3.72	3.61	0	(−0.3,0.2)	1987–2020	0	—	—	—	—	—	—	—	—
	female	3.12	2.48	−0.7 *	(−0.9,−0.6)	1987–2020	−0.7 *	—	—	—	—	—	—	—	—

^a^ Standardized to the WHO world standard population. ^b^ Years 1987 to 2020.—No joinpoints identified. Abbreviations: APC, annual percent change; AAPC, average annual percent change; * Significantly difference from zero (*p* < 0.05).

## Data Availability

The data presented in this study is available within the article and supplementary materials.

## Supplementary Materials

The following supporting information can be downloaded at: https://www.mdpi.com/article/10.3390/cancers15020476/s1, Figure S1: Trends in specific cancer ASMR in urban and rural populations in China, from 1987 to 2020; Figure S2: Local drift with net drift values for selected cancers mortality in rural and urban China, from 1987 to 2020; Figure S3: Parameter estimates of age, period, and cohort effects on selected cancer site mortality rate in China from 1987 to 2020; Table S1: Period Age-standardized mortality rates of selected cancer site in urban and rural China.

## Abbreviations

ASMR: age-standardized mortality rate; APC: age-period-cohort; CNHC: China’s National Health Commission; DALYs: disability-adjusted life years; ESCC: esophageal squamous cell carcinoma; ICD: International Classification of Diseases; NPC: Nasopharyngeal carcinoma; WSP: World Standard Population.

## References

[1] Chen W., Zheng R., Baade P.D., Zhang S., Zeng H., Bray F., Jemal A., Yu X.Q., He J. (2016). Cancer statistics in China, 2015. CA Cancer J. Clin..

[2] Sung H., Ferlay J., Siegel R.L., Laversanne M., Soerjomataram I., Jemal A., Bray F. (2021). Global Cancer Statistics 2020: GLOBOCAN Estimates of Incidence and Mortality Worldwide for 36 Cancers in 185 Countries. CA Cancer J. Clin..

[3] Zheng R., Zhang S., Zeng H., Wang S., Sun K., Chen R., Li L., Wei W., He J. (2022). Cancer incidence and mortality in China, 2016. J. Natl. Cancer Cent..

[4] Sun D., Cao M., Li H., He S., Chen W. (2020). Cancer burden and trends in China: A review and comparison with Japan and South Korea. Chin. J. Cancer Res..

[5] Zhou M., Wang H., Zeng X., Yin P., Zhu J., Chen W., Li X., Wang L., Wang L., Liu Y. (2019). Mortality, morbidity, and risk factors in China and its provinces, 1990–2017: A systematic analysis for the Global Burden of Disease Study 2017. Lancet.

[6] Sun D., Li H., Cao M., He S., Lei L., Peng J., Chen W. (2020). Cancer burden in China: Trends, risk factors and prevention. Cancer Biol. Med..

[7] Li L., Lu F., Zhang S. (1996). [Analysis of cancer modality and distribution in China from year 1990 through 1992—An epidemiologic study]. Zhonghua Zhong Liu Za Zhi.

[8] Qiu H., Cao S., Xu R. (2021). Cancer incidence, mortality, and burden in China: A time-trend analysis and comparison with the United States and United Kingdom based on the global epidemiological data released in 2020. Cancer Commun..

[9] Hanley A.J., Choi B.C., Holowaty E.J. (1995). Cancer mortality among Chinese migrants: A review. Int. J. Epidemiol..

[10] Yang D., Liu Y., Bai C., Wang X., Powell C.A. (2020). Epidemiology of lung cancer and lung cancer screening programs in China and the United States. Cancer Lett..

[11] Fang J.-Y., Dong H.-L., Wu K.-S., Du P.-L., Xu Z.-X., Lin K. (2015). Characteristics and prediction of lung cancer mortality in China from 1991 to 2013. Asian Pac. J. Cancer Prev..

[12] Lei S., Zheng R., Zhang S., Wang S., Chen R., Sun K., Zeng H., Zhou J., Wei W. (2021). Global patterns of breast cancer incidence and mortality: A population-based cancer registry data analysis from 2000 to 2020. Cancer Commun..

[13] Zhang L., Cao F., Zhang G., Shi L., Chen S., Zhang Z., Zhi W., Ma T. (2019). Trends in and predictions of colorectal cancer incidence and mortality in China from 1990 to 2025. Front. Oncol..

[14] Yang L., Zheng R., Wang N., Yuan Y., Liu S., Li H., Zhang S., Zeng H., Chen W. (2018). Incidence and mortality of stomach cancer in China, 2014. Chin. J. Cancer Res..

[15] Jinyao W., Nianping Z., Zhiqiang B., Zhenkun W. (2022). Age-Period-Cohort Analysis of Secular Trends of Cervical Cancer Incidence and Mortality in China, 1993—2017. Chin. Gen. Pract..

[16] Shi J.F., Canfell K., Lew J.B., Qiao Y.L. (2012). The burden of cervical cancer in China: Synthesis of the evidence. Int. J. Cancer.

[17] Guo P., Huang Z.L., Yu P., Li K. (2012). Trends in cancer mortality in China: An update. Ann. Oncol..

[18] Yang L., Parkin D.M., Li L., Chen Y. (2003). Time trends in cancer mortality in China: 1987–1999. Int. J. Cancer.

[19] Liu Q., Wang B., Kong Y., Cheng K.K. (2011). China’s primary health-care reform. Lancet.

[20] Xinhua China’s GDP Tops 100 Trillion Yuan in 2020. http://www.ecns.cn/news/2021-01-18/detail-ihafurte1836008.shtml.

[21] Feng R.M., Zong Y.N., Cao S.M., Xu R.H. (2019). Current cancer situation in China: Good or bad news from the 2018 Global Cancer Statistics?. Cancer Commun..

[22] Cai Y., Cui X., Su B.B., Wu S.Y. (2022). Changes in Mortality Rates of Major Chronic Diseases Among Populations Aged Over 60 Years and Their Contributions to Life Expectancy Increase—China, 2005–2020. China Cdc Weekly.

[23] Yue C., Shiyong W., Xiaoxu W., Ruixian W., Wenling Z. (2022). Improving the Quality of Vital Registration Data Through Multi-source Data Comparison. Chin. J. Health Stat..

[24] Ahmad O.B., Pinto C.B. Age Standardization of Rates: A New WHO Standard. https://www.researchgate.net/publication/284696312.

[25] National Cancer Institute Joinpoint Trend Analysis Software Version 4.9.1.0. https://surveillance.cancer.gov/joinpoint/.

[26] Zou Z., Cini K., Dong B., Ma Y., Ma J., Burgner D.P., Patton G.C. (2020). Time trends in cardiovascular disease mortality across the BRICS: An age-period-cohort analysis of key nations with emerging economies using the global burden of disease study 2017. Circulation.

[27] Condon J.R., Cunningham J., Barnes T., Armstrong B.K. (2004). Long-term trends in cancer mortality for Indigenous Australians in the Northern Territory. Med. J. Aust..

[28] Rosenberg P.S., Check D.P., Anderson W.F. (2014). A Web Tool for Age–Period–Cohort Analysis of Cancer Incidence and Mortality RatesSoftware for Cancer Rates and Trends. Cancer Epidemiol. Biomark. Prev..

[29] Qi H., Xi X. (2015). Why has China’s urbanization deviated from the goal of narrowing the urban-rural gap?—An analysis of the differences based on different stages of China’s economic development. Nanjing Soc. Sci..

[30] The Central People’s Government of China National Disposable Income per Capita in 2020. http://www.gov.cn/guoqing/2021-04/09/content_5598662.htm.

[31] Oberg M., Jaakkola M., Woodward A., Peruga A., Prüss-Ustün A. (2011). Worldwide burden of disease from exposure to second-hand smoke: A retrospective analysis of data from 192 countries. Lancet.

[32] Hamra G.B., Guha N., Cohen A., Laden F., Raaschou-Nielsen O., Samet J.M., Vineis P., Forastiere F., Saldiva P., Yorifuji T. (2014). Outdoor particulate matter exposure and lung cancer: A systematic review and meta-analysis. Environ. Health Perspect..

[33] Loomis D., Grosse Y., Lauby-Secretan B., El Ghissassi F., Bouvard V., Benbrahim-Tallaa L., Guha N., Baan R., Mattock H., Straif K. (2013). The carcinogenicity of outdoor air pollution. Lancet Oncol..

[34] Chen J.G., Zhang S.W. (2011). Liver cancer epidemic in China: Past, present and future. Semin. Cancer Biol..

[35] Tanaka M., Katayama F., Kato H., Tanaka H., Wang J., Qiao Y.L., Inoue M. (2011). Hepatitis B and C virus infection and hepatocellular carcinoma in China: A review of epidemiology and control measures. J. Epidemiol..

[36] Balakrishnan M., George R., Sharma A., Graham D.Y. (2017). Changing trends in stomach cancer throughout the world. Curr. Gastroenterol. Rep..

[37] Jemal A., Center M.M., DeSantis C., Ward E.M. (2010). Global Patterns of Cancer Incidence and Mortality Rates and TrendsGlobal Patterns of Cancer. Cancer Epidemiol. Biomark. Prev..

[38] Plummer M., Franceschi S., Vignat J., Forman D., De Martel C. (2015). Global burden of gastric cancer attributable to *Helicobacter pylori*. Int. J. Cancer.

[39] Bray F., Ferlay J., Soerjomataram I., Siegel R.L., Torre L.A., Jemal A. (2018). Global cancer statistics 2018: GLOBOCAN estimates of incidence and mortality worldwide for 36 cancers in 185 countries. CA Cancer J Clin.

[40] Mehta R.S., Song M., Nishihara R., Drew D.A., Wu K., Qian Z.R., Fung T.T., Hamada T., Masugi Y., da Silva A. (2017). Dietary patterns and risk of colorectal cancer: Analysis by tumor location and molecular subtypes. Gastroenterology.

[41] Jayasekara H., English D.R., Haydon A., Hodge A.M., Lynch B.M., Rosty C., Williamson E.J., Clendenning M., Southey M.C., Jenkins M.A. (2018). Associations of alcohol intake, smoking, physical activity and obesity with survival following colorectal cancer diagnosis by stage, anatomic site and tumor molecular subtype. Int. J. Cancer.

[42] Favoriti P., Carbone G., Greco M., Pirozzi F., Pirozzi R.E.M., Corcione F. (2016). Worldwide burden of colorectal cancer: A review. Updates Surg..

[43] Tang W.-R., Fang J.-Y., Wu K.-S., Shi X.-J., Luo J.-Y., Lin K. (2014). Epidemiological characteristics and prediction of esophageal cancer mortality in China from 1991 to 2012. Asian Pac. J. Cancer Prev..

[44] Zhao J., He Y.-T., Zheng R.-S., Zhang S.-W., Chen W.-Q. (2012). Analysis of esophageal cancer time trends in China, 1989–2008. Asian Pac. J. Cancer Prev..

[45] Liang H., Fan J.-H., Qiao Y.-L. (2017). Epidemiology, etiology, and prevention of esophageal squamous cell carcinoma in China. Cancer Biol. Med..

[46] Fan L., Strasser-Weippl K., Li J.-J., St Louis J., Finkelstein D.M., Yu K.-D., Chen W.-Q., Shao Z.-M., Goss P.E. (2014). Breast cancer in China. Lancet Oncol..

[47] Zhang M., Peng P., Wu C., Gong Y., Zhang S., Chen W., Bao P. (2019). Report of breast cancer incidence and mortality in China registry regions, 2008-2012. Zhonghua Zhong Liu Za Zhi [Chin. J. Oncol. ].

[48] Chen C., Sun S., Yuan J.-P., Wang Y.-H., Cao T.-Z., Zheng H.-M., Jiang X.-Q., Gong Y.-P., Tu Y., Yao F. (2016). Characteristics of breast cancer in Central China, literature review and comparison with USA. Breast.

[49] Grayson M. (2017). Bladder cancer. Nature.

[50] Isa F., Xie L.P., Hu Z., Zhong Z., Hemelt M., Reulen R.C., Wong Y.C., Tam P.C., Yang K., Chai C. (2013). Dietary consumption and diet diversity and risk of developing bladder cancer: Results from the South and East China case-control study. Cancer Causes Control.

[51] Bi W., Hayes R.B., Feng P., Qi Y., You X., Zhen J., Zhang M., Qu B., Fu Z., Chen M. (1992). Mortality and incidence of bladder cancer in benzidine-exposed workers in China. Am. J. Ind. Med..

[52] Tang L.-L., Chen W.-Q., Xue W.-Q., He Y.-Q., Zheng R.-S., Zeng Y.-X., Jia W.-H. (2016). Global trends in incidence and mortality of nasopharyngeal carcinoma. Cancer Lett..

[53] WHO Estimated Age-Standardized Incidence Rates (World) in 2020, All Cancers, Both Sexes, All Ages. https://gco.iarc.fr/today/online-analysis-map.

[54] Kwok H., Wu C.W., Palser A.L., Kellam P., Sham P.C., Kwong D.L., Chiang A.K. (2014). Genomic diversity of Epstein-Barr virus genomes isolated from primary nasopharyngeal carcinoma biopsy samples. J. Virol..

[55] Chang E.T., Liu Z., Hildesheim A., Liu Q., Cai Y., Zhang Z., Chen G., Xie S.H., Cao S.M., Shao J.Y. (2017). Active and Passive Smoking and Risk of Nasopharyngeal Carcinoma: A Population-Based Case-Control Study in Southern China. Am. J. Epidemiol..

[56] Ho J.H.C., Huang D.P., Fong Y.Y. (1978). Salted Fish and Nasopharyngeal Carcinoma in Southern Chinese. Lancet.

[57] Hua Z.Z., Ying L.C., Ye R.H., Shaohui L. (2022). A study on the trends of cervical cancer incidence and mortality among Chinese women during 2003-2018. Chin. J. Dis. Control.

[58] Zeng H., Chen W., Zheng R., Zhang S., Ji J.S., Zou X., Xia C., Sun K., Yang Z., Li H. (2018). Changing cancer survival in China during 2003–15: A pooled analysis of 17 population-based cancer registries. Lancet Glob. Health.

[59] Henley S.J., Singh S.D., King J., Wilson R.J., O’Neil M.E., Ryerson A.B. (2017). Invasive Cancer Incidence and Survival—United States, 2013. MMWR Morb. Mortal. Wkly. Rep..

