# Supplementary material for: Changing Patterns in Cancer Mortality from 1987 to 2020 in China
Source: Cancers (Basel). 2023 Jan 12;15(2):476. doi: 10.3390/cancers15020476 (PMC9856369; doi:10.3390/cancers15020476)
Supplement: Supplementary file 1 [file cancers-15-00476-s001.zip › cancers-2104590-supplementary.pdf]

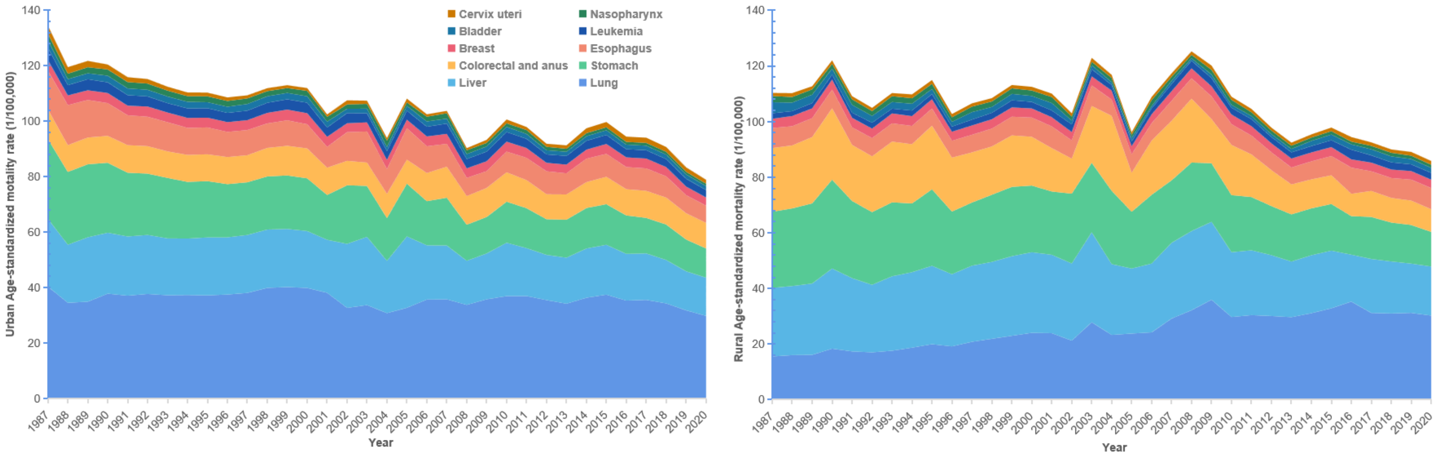

Figure S1. Trends in specific cancer ASMR in urban and rural populations in China, from 1987 to 2020.

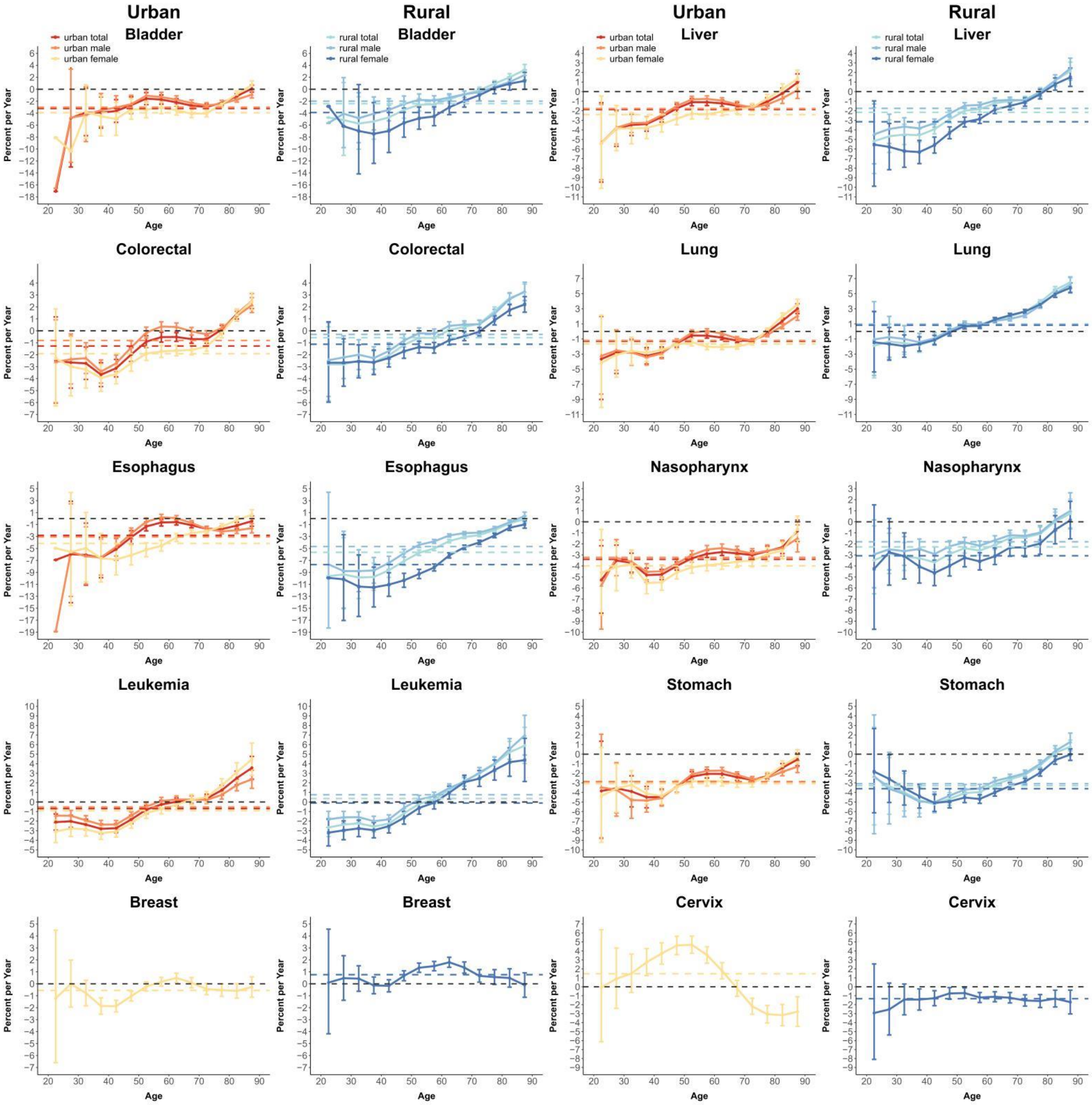

Figure S2. Local drift with net drift values for selected cancers mortality in rural and urban China, from 1987 to 2020

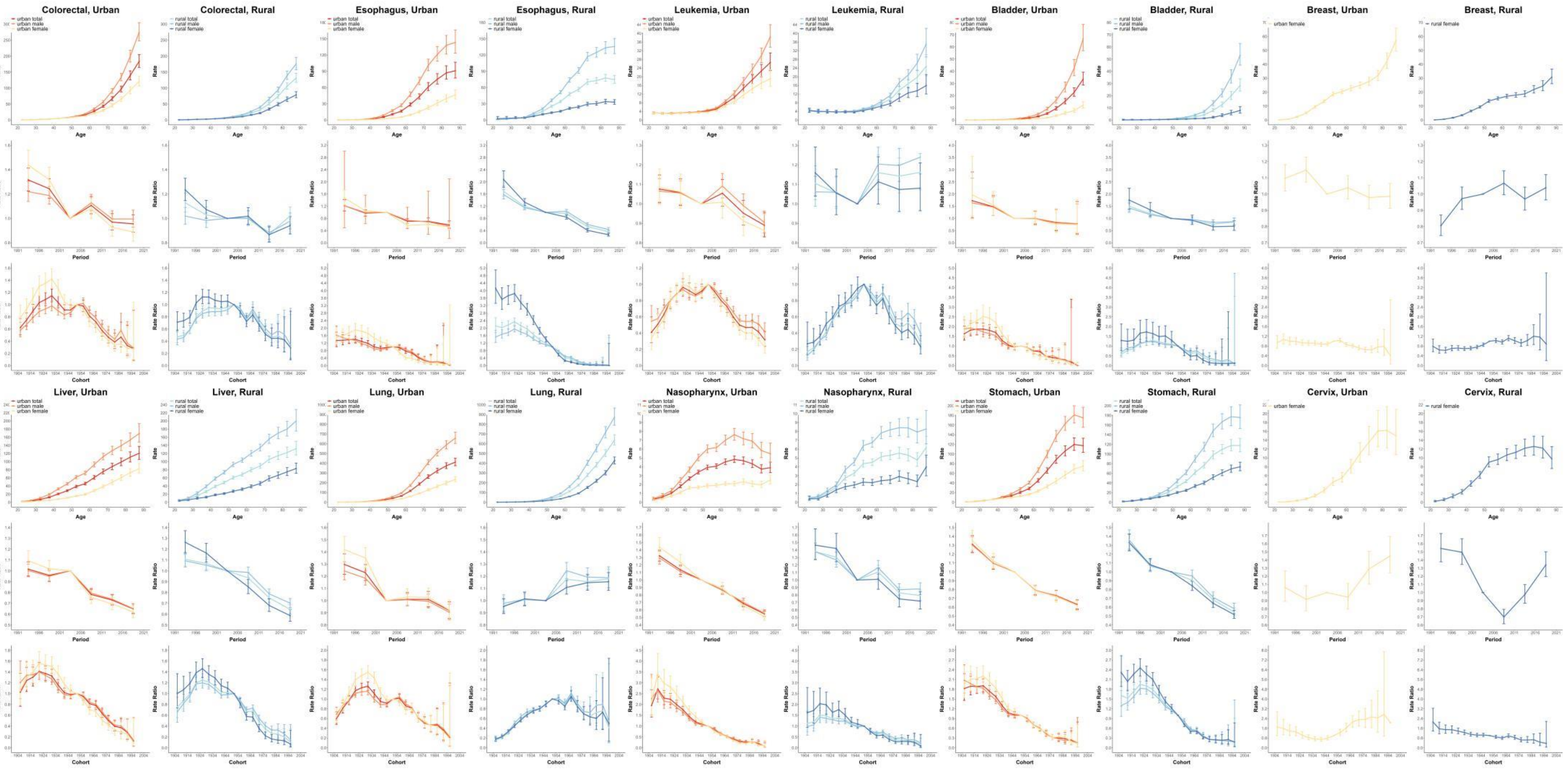

Figure S3. Parameter estimates of age, period, and cohort effects on selected cancer site mortality rate in China from 1987 to 2020.

Table S1. Period Age-standardized mortality rates of selected cancer site in urban and rural China.

Area-Sex	Cancer Site	Period Age-Standardized Mortality Rates (1/100,000)						
		1987-1991	1992-1996	1997-2001	2002-2006	2007-2011	2012-2016	2017-2020
Rural-male	All cancers	163.86	161.74	162.04	167.40	192.22	157.92	149.04
	Bladder	2.06	2.08	2.02	1.92	2.21	1.97	2.22
	Breast	0.00	0.00	0.00	0.00	0.00	0.00	0.00
	Cervix uteri	0.00	0.00	0.00	0.00	0.00	0.00	0.00
	Colorectal and anus	7.72	7.22	8.19	8.02	9.26	8.04	9.22
	Leukemia	3.85	3.69	3.73	3.46	4.08	3.72	3.65
	Liver	38.17	39.03	40.47	39.31	39.82	30.95	27.80
	Lung	25.13	27.83	33.01	35.34	47.82	44.69	44.97
	Nasopharynx	2.72	2.69	2.76	2.17	2.22	1.82	1.83
	Esophagus	30.42	28.55	23.99	25.85	27.90	16.36	13.73
	Stomach	38.73	35.26	31.89	33.19	31.32	23.69	20.03
Rural–female	All cancers	91.25	88.51	88.75	86.05	89.81	78.48	74.84
	Bladder	0.53	0.67	0.58	0.50	0.51	0.44	0.49
	Breast	3.58	3.65	4.70	4.48	5.13	4.57	4.88
	Cervix uteri	5.79	4.00	4.71	2.17	2.16	3.30	3.96
	Colorectal and anus	5.99	5.68	5.64	5.31	5.78	5.09	5.62
	Leukemia	3.21	3.20	2.93	2.81	2.94	2.75	2.51
	Liver	14.34	14.88	15.95	13.78	12.85	10.39	9.18
	Lung	9.21	10.69	13.33	13.33	16.93	17.95	17.90
	Nasopharynx	1.25	1.14	1.29	0.84	0.81	0.63	0.63
	Esophagus	16.62	14.95	11.45	11.82	10.46	5.42	4.18
	Stomach	19.89	17.33	16.15	15.74	12.88	10.06	8.16
Urban-male	All cancers	191.64	181.06	178.58	171.03	167.44	160.36	147.38
	Bladder	3.91	3.96	3.93	2.79	3.05	2.68	2.63
	Bladder	0.00	0.00	0.00	0.00	0.00	0.00	0.00
	Breast	0.00	0.00	0.00	0.00	0.00	0.00	0.00
	Colorectal and anus	10.81	11.09	11.82	10.73	12.81	11.48	12.06
	Leukemia	4.44	3.90	4.14	3.85	4.12	3.65	3.37
	Liver	32.90	30.75	30.34	33.28	26.59	25.66	22.56
	Lung	51.73	53.75	55.66	48.62	51.91	51.80	48.16
	Nasopharynx	3.10	3.09	2.66	2.52	2.19	1.69	1.44
	Esophagus	18.89	15.45	13.56	15.68	11.79	12.65	11.13
	Stomach	36.44	29.62	26.18	25.31	20.72	19.82	17.11
Urban–female	All cancers	108.52	98.91	100.04	88.98	90.08	84.10	77.30
	Bladder	1.17	1.10	1.05	0.76	0.81	0.68	0.67
	Breast	6.88	6.87	7.34	6.35	6.80	6.37	6.27
	Cervix uteri	4.10	2.62	1.95	2.08	2.01	3.13	3.27

	Colorectal and anus	9.20	8.69	9.05	7.43	8.60	7.43	7.28
	Leukemia	3.48	3.06	3.07	2.92	2.87	2.65	2.37
	Liver	12.04	11.31	11.22	11.69	9.16	8.76	7.78
	Lung	23.49	23.20	24.97	19.05	21.62	20.93	18.74
	Nasopharynx	1.37	1.19	1.10	0.91	0.78	0.56	0.47
	Esophagus	7.69	5.28	4.44	5.34	3.15	3.89	3.35
	Stomach	16.51	13.31	11.94	11.32	9.08	8.51	7.16
